# Impact of mobile Internet use on health-seeking behaviors: evidence from China

**DOI:** 10.3389/fpubh.2024.1403877

**Published:** 2024-06-20

**Authors:** ChenLei Lin, Hong Lin

**Affiliations:** School of Public Administration and Law, Fujian Agriculture and Forestry University, Fuzhou, China

**Keywords:** health-seeking behaviors, mobile Internet, health information, health heterogeneity, China

## Abstract

**Introduction:**

Although health-seeking behaviors are crucial to China’s healthcare delivery system, the influence of mobile Internet use in this context remains under-explored. This study aimed to comprehensively explore the influence of mobile Internet use on health-seeking behaviors, and meticulously examined the heterogeneity in health outcomes associated with the intersection between mobile Internet use and health-seeking behaviors.

**Methods:**

We used nationally representative data derived from the China Family Panel Studies. Given that individuals typically make the decision to use mobile Internet autonomously, an instrumental variable regression methodology was adopted to mitigate potential selection biases.

**Results:**

Our findings revealed that mobile Internet use significantly promoted self-medication and adversely affected the use of primary care facilities among Chinese adults. Furthermore, our findings highlighted the heterogeneous effects of mobile Internet use across diverse health demographic groups.

**Conclusion:**

These findings underscore the importance of strategic planning and utilizing mobile Internet resources to steer individuals toward more appropriate healthcare-seeking behaviors.

## 1 Introduction

According to Billari et al. (1), a new era in the digital revolution is emerging. Moreover, information and communication technologies (ICTs) are at the forefront of significant transformations in the distribution and accessibility of health and medical information. Fox and Duggan (2) reported that many Internet users actively seek health information online. This transition facilitates individuals in acquiring knowledge regarding their health, addressing health challenges, making informed health decisions, and adopting behavioral changes (3, 4), which has significantly narrowed the knowledge and power gap between healthcare professionals and the general populace (5). A nationwide survey by Wang et al. (6) revealed that approximately 33.2% of Chinese adults actively sought health information via the Internet. Following the outbreak of COVID-19, the “China Internet Development Report 2022” revealed that, as of December 2022, the number of users participating in health-related Internet activities in China had increased to 363 million, accounting for 34% of the total Chinese Internet users. Owing to their importance, these developments have garnered increasing attention from policy-makers and researchers in developing countries.

ICTs have significantly amplified the dissemination of medical information, leading to profound changes in the volume, quality, and scope of accessible information (7). This, in turn, has had a consequential impact on individuals’ health-seeking behaviors (8). Within the medical marketplace, “information represents a valuable commodity”; consequently, transformations in the dynamics of information supply and demand are set to have a compelling “inducing effect” on the health-seeking behaviors of individuals (9, 10). A strand of current research has been dedicated to exploring the nexus between ICTs and health-seeking behaviors. However, elucidation of the precise nature of this relationship is ongoing. The research conducted by Lee et al. (11), which employs data derived from New York City, suggests that Internet use can markedly influence the health-related attitudes and behaviors of a significant proportion of the population, as well as the management of chronic diseases. Nevertheless, some scholars, such as Takahashi et al. (12) and Zwijnenberg et al. (13), have posited that patients’ interest in online comparative healthcare information seldom translates into tangible changes in their health-related behaviors.

Although the existing academic literature has extensively explored the role of ICTs and the intricate relationship between ICTs and health-seeking behaviors, the nexus between mobile Internet use and health-seeking behaviors has not been explicitly analyzed, particularly concerning the heterogeneous nature of health impacts attributed to mobile Internet use. Among the myriad of ICTs, the proliferation of the mobile Internet has been remarkable recently, which is largely attributed to the widespread adoption of mobile devices, such as smartphones and tablets (3). Compared with broadband Internet, mobile Internet allows users to access up-to-date information irrespective of their geographical location. Furthermore, the mobile Internet allows users to install and operate various mobile applications (apps) for several purposes, as highlighted by Kirwan et al. (14) and Soikkeli et al. (15). Ozdalga et al. (16) and Xia et al. (17) emphasize that modern mobile phones are equipped with enhanced memory, faster processors, larger interfaces, sophisticated navigation systems, and a comprehensive array of functionalities and features, including more streamlined access to the Internet. We hypothesized that a comprehensive and nuanced understanding of the nexus between mobile Internet use and health-seeking behaviors is important to offer crucial insights for policymakers regarding the types of initiatives that could be employed to enhance the constructive guidance of health-seeking behaviors.

A significant segment of scholarly literature on the role of ICTs within the healthcare system has primarily focused on the implications of broadband Internet (7, 18, 19). Nevertheless, a scant number of empirical studies have systematically explored the impact of mobile Internet on health-seeking behaviors. Descriptive statistical analysis and logistic regression techniques, as employed in various studies, have been instrumental in investigating the multi-faceted effects of ICTs on healthcare systems (7, 20); however, they lacked scientific methods to infer causal relationships, thereby failing to accurately capture the genuine impact of policy interventions. Several studies, including those by Zhou et al. (21), have posited that patients frequently use the Internet to identify diseases that correspond to their symptoms and assess their health status following the onset of these symptoms, subsequently informing their medical decision-making processes. Therefore, we aimed to provide more evidence by investigating the heterogeneous effects of mobile Internet use among different health groups.

## 2 Literature review and research hypotheses

The foundational behavioral model of healthcare utilization, developed by Andersen (22), delineates health-seeking behaviors as a pivotal behavioral element that fundamentally supports the utilization of healthcare services (23). An effective strategy for understanding health service utilization concerns examining health-seeking behaviors, which involves two steps: First, when residents are sick, they decide whether to seek healthcare or not, including the option of self-medication. If they choose to seek healthcare, the second step is deciding between visiting primary care facilities (PCFs) or higher-level hospitals (24–26). The inefficient utilization of healthcare resources presents a perennial challenge to global healthcare systems and is particularly pronounced in China, underscoring the urgency to understand individuals’ health-seeking behaviors. In China, the healthcare delivery system is predominantly hospital-centric and fragmented. Given that patients often harbor doubts regarding the quality of care provided by PCFs, it is common for them to sidestep local PCFs and directly seek services from higher-level hospitals, even for minor and common ailments (27, 28). Consequently, understanding and analyzing health-seeking behaviors among Chinese residents is a priority, and the determinants of these behaviors have attracted considerable attention from the scholarly community (29, 30).

Recently, the wide-spread availability of mobile Internet globally has played a crucial role in enhancing accessibility to medical information, bolstering social support networks, and forging new pathways for interactions between patients and healthcare professionals (31, 32). Furthermore, compared with the offline world, mobile Internet presents numerous patient benefits, including enhanced convenience, increased time efficiency, and reduced space- and time-related constraints (33). Therefore, we posit that mobile Internet use may significantly influence health-seeking behaviors. The advent of mobile Internet has empowered individuals to acquire healthcare knowledge through diverse platforms, including online medical lectures and healthcare-focused mobile applications (14, 34). These digital platforms provide exhaustive diagnostic and therapeutic information on a wide spectrum of prevalent diseases by incorporating essential definitions and symptoms, strategies for medication administration, and detailed contraindications (35). If a patient determines that the ailment is common and that their health status is stable, they are likely to procure medications and engage in self-diagnosis in accordance with the online treatment protocol. Conversely, if the assessment is incorrect, the individual will likely opt for a consultation with a physician.

However, theoretically, access to a more extensive array of information should ostensibly culminate in more informed decision-making. Nevertheless, the advantages of increasing information within the healthcare sector are multi-faceted and complex (36). Given the diverse array of sources of mobile Internet information, this multiplicity could intensify the fragmentation of individual disease knowledge, potentially heightening individuals’ dependence on authoritative medical institutions for accurate information. Consequently, individuals, especially those in poor health conditions, may frequently bypass local PCFs and opt for services from higher-tier hospitals, driven by a potential lack of trust in the quality of care provided at PCFs. Another possibility is that individuals in good health conditions may be inclined to overestimate the negative outcomes of their health conditions, consequently exacerbating the expected loss of utility associated with misdiagnosis. This tendency can be attributed to the fact that medical information obtained from the mobile Internet predominantly concentrates on delivering universal and basic content, frequently neglecting to address the probability of potential health consequences. Based on the foregoing analysis, this study proposed the following hypotheses:

*Hypothesis 1*: Mobile Internet use substantially enhances the propensity for self-medication among Chinese residents.

*Hypothesis 2*: Chinese mobile Internet users are less likely to seek medical care from PCFs.

*Hypothesis 3*: Mobile Internet use significantly increases the inclination toward self-treatment among residents with good health conditions while simultaneously decreasing the propensity for visits to PCFs among residents regardless of their health status.

## 3 Materials and methods

### 3.1 Data

The foundational statistical data in our analysis originated from the 2020 cycle of the China Family Panel Studies. This comprehensive biennial longitudinal survey offers a nationally representative snapshot of Chinese households. This dataset is optimally suited to our research objectives, offering a comprehensive collection of data related to mobile Internet use, in addition to detailed records encompassing health-seeking behaviors, health status, and demographic characteristics. The dataset encompasses a sample of approximately 14,960 households spanning 25 provinces, cities, and autonomous regions, representing approximately 94.5% of the country’s population (37).

Owing to the availability of daily Internet usage data exclusively in the most recent survey, our analysis was confined to data from 2020. The 2020 China Family Panel Studies database comprised three distinct sections: family, adult, and children databases. In response to the specific requirements of our study, we analyzed the data from the adult and family databases. Initially, we aligned and integrated data from the adult and family databases and subsequently eliminated redundant samples. Thereafter, the data was refined by excluding entries that lacked essential variable information. Ultimately, after data selection and cleansing, 22,496 observations were selected. The sample size used for the regression analyses was subject to variation, contingent on the specific model specifications and data availability for pertinent variables.

### 3.2 Definitions of variables

#### 3.2.1 Dependent variables

Health-seeking behaviors served as the dependent variables. It has been defined as any action undertaken by individuals who perceive themselves to have a health problem or to be ill for the purpose of finding an appropriate remedy (38). Based on the behavioral model of healthcare utilization, health-seeking behaviors encompass the pursuit of self-medication during illness and the selection of health facilities. The primary measure of health-seeking behavior is self-medication use, which is defined as the practice of individuals treating their ailments and conditions with medications or remedies without professional supervision (39, 40). In the present study, the corresponding question was as follows: “Have you consulted a physician regarding any illnesses experienced within the previous 2 weeks?” A dichotomous outcome variable was used, taking a value of “1” if the individual consulted a physician and “0” if the individual did not consult a physician but there was a purchase of medication with an expenditure greater than 0. Additionally, based on studies by Minhas et al. (41) and Zhou et al. (26), the answer to the question “To which kind of facilities do you usually go to seek health services when you are sick?” was used as the type of health care facilities that residents usually approach when seeking health services. This aspect effectively mirrors residents’ choice of different levels of healthcare institutions during instances of illness, an occurrence commonly denoted as health-seeking behavior (26). The dependent variable was delineated as a binary variable contingent on the level of healthcare facility chosen by the individual. In this research context, the variable was assigned a value of “1” if a resident opted for PCFs, encompassing entities such as community health centers, community health service stations, township health centers, and village clinics. Conversely, the variable was assigned a value of “0” when a resident opted for hospital care, encompassing both general hospitals that provide comprehensive treatment for various conditions and specialist hospitals that focus on specific medical issues.

#### 3.2.2 Independent variables

According to studies by Khalaila and Vitman-Schorr (42) and Lu and Kandilov (43), our variable of interest, mobile Internet use, refers to the use of mobile devices, such as smartphones and tablet computers, to access the Internet. For this measure, a value of “1” was used to indicate instances where respondents incorporated mobile Internet use into their daily routines through any mobile device, such as smartphones or tablet computers. To enhance the robustness of the analysis, we adopted for an alternative measure of mobile Internet use, which was predicated on quantifying the duration of mobile Internet use. It was determined based on the response to the question, “On average, how many hours per day do you use your mobile device to access the Internet?” This measure aimed to quantify the intensity of mobile Internet engagement. This measure was selected to complement the principal dichotomous metric of mobile Internet use, thereby substantially enriching our understanding of the complex and varied nature of mobile Internet engagement among respondents.

#### 3.2.3 Controlled variables

Based on the research by Andersen (22) and Clewley et al. (23), we included four categories of control variables: (1) predisposing factors, encompassing a spectrum of demographic variables, such as age, sex, marital status, level of education, and geographical location of residence; (2) enabling factors, encompassing annual household income and health insurance coverage; (3) need factors, encompassing self-reported health, chronic diseases, smoking habits, and frequent alcohol consumption (at least three times per week); and (4) supply-side factors, encompassing the ratio of PCFs per 1,000 residents as a significant determinant potentially influencing individuals’ health-seeking behaviors and their preferences for healthcare providers (28, 44, 45). Detailed definitions and explanations of these categories are presented in Table 1.

**Table 1:** Descriptive statistics.

Variables	Definition	Full sample		Users	Non-users	Diff.
		Mean	SD	Mean	Mean	
*Dependent variables*						
Self-medication	Yes = 1, No = 0	0.637	0.481	0.441	0.264	0.177***
PCF choice	Yes = 1, No = 0	0.570	0.495	0.530	0.645	0.115***
*Independent variables*						
Mobile Internet use	Yes = 1, No = 0	0.655	0.475	–	–	–
Daily Internet use	Hours	1.967	2.681	–	–	–
*Control variables*						
Age	Years	45.200	16.384	38.287	58.322	20.035***
Age squared	Years	2311.450	1544.731	1650.162	3566.650	1916.488***
Sex	Male = 1, Female = 0	0.503	0.500	0.514	0.481	−0.033***
Marriage status	Married = 1, Other = 0	0.773	0.419	0.730	0.856	0.126***
Education	Years	8.977	4.755	10.824	5.470	−5.354***
Rural resident	Rural = 1, Urban = 0	0.718	0.450	0.665	0.818	0.152***
Health insurance	Insured = 1, Other = 0	0.899	0.301	0.892	0.912	0.020***
Family income	Chinese Yuan (log)	11.152	1.090	11.360	10.756	−0.604***
Self-reported health	Excellent = 1, Very good = 2, Good = 3, Fair = 4, Poor = 5	2.891	1.188	2.732	3.192	0.460***
Chronic disease	Yes = 1, No = 0	0.143	0.351	0.104	0.218	0.114***
Smoking	Yes = 1, No = 0	0.275	0.447	0.274	0.277	0.003
Drinking	Yes = 1, No = 0	0.126	0.332	0.114	0.149	0.034***
Number of PCFs per 1,000 residents	Continuous variables	0.725	0.237	0.718	0.737	0.020***

Significance levels are obtained from *t*-tests. The descriptive statistics for provincial dummies are not presented. *p < 0.1; **p < 0.05; ***p < 0.01. SD, standard deviation; PCFs, primary care facilities.

### 3.3 Empirical strategies

To explore the impact of mobile Internet use on health-seeking behaviors, we used logistic regression (logit) analysis to estimate the following model:


(1) $$\log\left(Y_{1i}\right)=\alpha_{0}+\alpha_{1}\mathrm{Interne}t_{i}+\alpha_{2}X_{i}+\varepsilon_{i}$$

(2) $$\log\left(Y_{2i}\right)=\alpha_{0}+\alpha_{1}\mathrm{Interne}t_{i}+\alpha_{2}X_{i}+\varepsilon_{i}$$

In Equations (1) and (2), the subscript i represents the i−th individual respondent. The dependent variable $Y_{1}$ is the self-medication choice of respondent i , and $Y_{2}$ is the choice of medical facilities. The key independent variable is the status of mobile Internet use. Additionally, a comprehensive vector of control variables $X_{i}$ was included in the model, and the error term $\varepsilon_{i}$ was used to account for unobserved heterogeneity.

Using a logit model to estimate the effect of mobile Internet use on health-seeking behaviors is associated with challenges, such as endogeneity and omission of relevant variables. For instance, mobile Internet users are often characterized by higher levels of education and income, which may correlate with a more advanced understanding of health information and greater financial ability. Such factors can enhance self-medication tendencies and inclinations to opt for healthcare services from higher-tier hospitals, potentially leading to biased estimates. To attain reasonable and consistent regression outcomes regarding the impact of Internet development on health, we used the instrumental variable (IV) method.

Building on the findings of prior research (1, 46), the proportion of plain areas and average Internet usage rated as IVs were used to examine mobile Internet usage. Regions characterized by a higher prevalence of plains exhibit relatively fewer challenges in Internet infrastructure development, thereby enhancing mobile Internet accessibility in these locales. Conversely, in regions with scanty plain areas, the more complex terrain poses unfavorable geographical conditions for constructing diverse Internet infrastructure. Owing to data availability, we selected data from various Chinese provinces as IVs. Specifically, we used the proportion of plain areas within these provinces (denoted as “Land”) as the IV to gauge mobile Internet usage. However, an elevated average mobile Internet usage rate in urban areas could potentially amplify individual access to mobile Internet, which is attributable to shared local Internet infrastructure and cultural influences. Consequently, the mean mobile Internet usage rate among other respondents within the same city, excluding the respondent in question, was used as a secondary IV. These two IVs demonstrate a significant correlation with mobile Internet use; however, they are not presumed to directly affect an individual’s healthcare-seeking behaviors, except through mobile Internet. To ensure the robustness of our analysis, we used a range of methodologies, including IV-probit, extended regression models, and propensity score matching, to thoroughly ascertain and validate the reliability of our findings.

## 4 Results

### 4.1 Descriptive statistics

Table 1 presents the definitions and a comprehensive summary of the statistical analyses conducted for both dependent and independent variables. The sample data showed that the mean age of the respondents was approximately 45 years, indicating that mobile Internet users were markedly younger than non-users. Furthermore, most mobile Internet users were educated, residents of urban areas, and had higher family incomes. Regarding health-seeking behaviors, a greater proportion of mobile Internet users opted for self-medication than did non-users when ill (0.441 versus 0.264, *p* < 0.01). Notably, approximately 53% of mobile Internet users chose PCFs for health services, compared with 64.5% of non-users.

### 4.2 Effects of mobile Internet use on health-seeking behaviors

Table 2 presents the estimation results derived from the logit regression analysis, effectively determining the impact of mobile Internet use on residents’ health-seeking behaviors. In Model 1, the analysis was conducted without including any control variables. In Model 2, an augmented logit regression model incorporating a set of additional covariates was used to rigorously evaluate the impact of mobile Internet use on self-medication practices and preferences for PCFs. Regarding self-medication, the odds ratios (ORs) for mobile Internet use consistently exceeded 1 and remained statistically significant even when controlling for various covariates. This implies that the OR of residents opting for self-medication after using mobile Internet was 33.0%, a rate notably higher than that of non-users (OR: 2.157, *p* < 0.01 in Model 1; OR: 1.330, *p* < 0.01 in Model 2). This finding underscores the fact that mobile Internet use significantly increases the propensity of individuals to opt for self-medication. Consequently, this substantiates Hypothesis 1, affirming the positive correlation between mobile Internet use and the inclination toward self-medication. Regarding the choice of PCFs, the ORs for mobile Internet use were consistently below 1, indicating a statistically significant effect after adjusting for potential confounding covariates (OR: 0.631, *p* < 0.01 in Model 1; OR: 0.844, *p* < 0.01 in Model 2). Therefore, mobile Internet use significantly influenced residents’ decisions to opt for high-level hospitals, thereby supporting Hypothesis 2.

**Table 2:** Impact of mobile Internet use on health-seeking behaviors.

	Self-medication		PCF choice	
	Model 1	Model 2	Model 1	Model 2
Mobile Internet use	2.157^***^ (0.057) [0.769]	1.330^***^ (0.082) [0.285]	0.631^***^ (0.029) [−0.461]	0.844^***^ (0.042) [−0.170]
Age		1.014 (0.013)		1.014^**^ (0.006)
Age squared		1.000 (0.000)		1.000^***^ (0.000)
Sex		0.981 (0.078)		1.130^***^ (0.037)
Marriage status		0.769^***^ (0.085)		1.096^**^ (0.044)
Education		1.038^***^ (0.008)		0.927^***^ (0.004)
Rural resident		0.706^***^ (0.076)		2.206^***^ (0.036)
Health insurance		0.855 (0.106)		0.904^**^ (0.050)
Family income		0.952^*^ (0.028)		0.897^***^ (0.017)
Self-reported health				
Very good		1.483^**^ (0.173)		1.134^**^ (0.052)
Good		1.327^*^ (0.145)		1.014 (0.045)
Fair		1.349^*^ (0.159)		1.053 (0.063)
Poor		1.168 (0.150)		0.661^***^ (0.061)
Chronic disease		0.356^***^ (0.076)		0.591^***^ (0.047)
Smoking		1.216^**^ (0.085)		0.981 (0.041)
Drinking		1.311^***^ (0.099)		1.039 (0.048)
Number of PCFs per 1,000 residents		0.612^***^ (0.136)		2.099^***^ (0.073)
Constant	0.364^***^ (0.044)	1.337 (0.648)	1.792^***^ (0.024)	3.086^***^ (0.246)
R-squared	0.025	0.086	0.009	0.085
Observations (*N*)	5,798	5,357	22,496	21,049

Cells represent odds ratios (robust standard errors) [coefficients]. ^*^p < 0.1; ^**^p < 0.05; ^***^p < 0.01. PCFs, primary care facilities.

To address potential endogeneity concerns, we used “Land” and “average Internet usage” as IVs for mobile Internet use. Results of the two-stage least squares estimation are presented in Table 3. The first stage of the regression analysis showed that both IVs had a constructive impact on self-medication and the preference for PCFs. Importantly, the Kleibergen–Paap and Wald F-statistic attained substantial values of 61.742 (*p* < 0.01) and 192.553 (*p* < 0.01), respectively, firmly repudiating the null hypothesis of weak instruments. Furthermore, the results of the Hansen J test for instrument exogeneity yielded values of 0.860 (*p* > 0.10) and 0.160 (*p* > 0.10), corroborating the veracity and appropriateness of the chosen IVs within the framework of our analysis. Considering the endogeneity of mobile Internet use, its effect on health-seeking behaviors remains significant. An increase of one standard deviation (SD) in mobile Internet use enhances the probability of self-medication by 0.293 SDs and diminishes the likelihood of opting for PCFs by 0.638 SDs. These values are substantially larger than the logit estimates, as shown in Table 2. This discrepancy suggests that the logit estimates may exhibit a downward bias attributable to the endogeneity within the model.

**Table 3:** Tests for the validity of the two instrumental variables and the 2SLS estimates.

	Self-medication 2SLS		PCF choice 2SLS	
	First stage (1)	Second stage (2)	First stage (3)	Second stage (4)
Land	0.121^***^ (0.028)		0.081^***^ (0.013)	
Average Internet use	0.377^***^ (0.038)		0.311^***^ (0.017)	
Mobile Internet use		0.293^**^ (0.114)		−0.638^***^ (0.076)
Constant	0.245^***^ (0.086)	0.459^***^ (0.120)	0.238^***^ (0.040)	0.981^***^ (0.064)
Controls	Yes	Yes	Yes	Yes
Hansen J statistic	0.860		0.160	
Kleibergen–Paap F-statistic	61.742^***^		192.553^***^	
Observations (*N*)	5,287	5,287	20,763	20,763

The cells represent the coefficients (robust standard errors). ^***^p < 0.01, ^**^p < 0.05, ^*^p < 0.1. PCF, primary care facility; 2SLS, Two-Stage Least Squares.

### 4.3 Robustness checks

The first check for robustness focused on the alternative metrics for mobile Internet use. In Table 4, we provide the logit model estimates. We conducted an in-depth analysis of the influence of mobile Internet use on health-seeking behavior. We employed a proxy variable representing daily mobile Internet use, distinct from general mobile Internet use, as an independent variable. The findings indicate a positive effect of daily mobile Internet use on self-medication and a negative impact on PCF selection, suggesting that the substantial effect of daily mobile Internet use on health-seeking behaviors remains consistent across various measures of mobile Internet use.

**Table 4:** Robustness checks: alternative measures of mobile Internet use.

	Self-medication (odd ratios)		PCF choice (odd ratios)	
	Model 1	Model 2	Model 1	Model 2
Daily Internet usage	1.146^***^ (0.013) [0.137]	1.042^***^ (0.015) [0.041]	0.906^***^ (0.005) [−0.099]	0.949^***^ (0.007) [−0.052]
Constant	0.455^***^ (0.033)	1.208 (0.488)	1.609^***^ (0.017)	3.597^***^ (0.246)
Controls	No	Yes	No	Yes
R-squared	0.021	0.085	0.012	0.086
Observations (*N*)	5,798	5,357	22,440	21,049

Cells represent odds ratios (robust standard errors) [coefficients]. ^*^p < 0.1; ^**^p < 0.05; ^***^p < 0.01. PCF, primary care facility.

As detailed in Table 5, a series of tests were performed to ascertain the robustness of our results against various specifications. In Panels A and B of the table, we reassess the influence of mobile Internet use on health-seeking behaviors using the IV-probit and extended regression model methodologies, respectively. Acknowledging that sample selection bias might contribute to biased estimates, we ultimately used propensity score matching to estimate the average treatment effects on the treated group. To ensure that the various matching methods employed do not unduly impact the estimation outcomes, we applied multiple techniques, including the nearest-neighbor (1, 3), kernel, and radius matching methods. As shown in Panel C of Table 5, the average treatment effect before matching significantly exceeded the post-matching effect, highlighting the critical role of addressing selection bias in preventing over-estimation of the influence of mobile Internet use on health-seeking preferences. Remarkably, the estimation results obtained through the diverse matching methods remained inherently consistent, thereby underscoring the robustness of the choice of matching methodology.

**Table 5:** Robustness checks: alternative specifications.

	Self-medication	PCF choice
	Panel A: IV-probit	
Mobile Internet use	0.786 (0.289) ^***^	−1.497 (0.132) ^***^
Constant	−0.171 (0.335)	1.159 (0.139) ^***^
Controls	Yes	Yes
Observations (*N*)	5,287	20,763
	Panel B: ERMs	
Mobile Internet use	0.397 (0.136) ^***^	−0.701 (0.072) ^***^
Constant	0.040 (0.305)	0.942 (0.139) ^***^
Controls	Yes	Yes
Observations (*N*)	5,287	20,763
	Panel C: PSM	
Before matching ATT	0.177 (0.013) ^***^	−0.114 (0.007) ^***^
Nearest-neighbor matching (1:3) ATT	0.065 (0.037) ^*^	−0.053 (0.020) ^***^
Radius matching ATT	0.073 (0.031) ^**^	−0.078 (0.016) ^***^
Kernel matching ATT	0.064 (0.032) ^**^	−0.070 (0.016) ^***^
Observations (*N*)	5,357	21,049

The cells represent the coefficients (robust standard errors). ^***^p < 0.01, ^**^p < 0.05, ^*^p < 0.1. PSM, propensity score matching; IVs, instrumental variables; PCF, primary care facility; ERM, extended regression model; ATT, average treatment effects on the treated group.

### 4.4 Heterogeneous effects

The analysis conducted in this study revealed that mobile Internet use significantly increased the likelihood of individuals seeking self-medication, causing an increased influx of patients to more advanced medical facilities. However, it is worth noting that the influence of mobile Internet use on health-seeking behaviors exhibits heterogeneity among diverse health groups. To verify Hypothesis 3, a comprehensive analysis of the various influences on groups with disparate health conditions was performed. Self-reported health status, a frequently employed surrogate in health research (47), constitutes the primary health metric used in this study. Self-reported health status is a comprehensive reflection of an individual’s overall health status and a reliable metric for assessing morbidity, physical well-being, and the decline in functional capacity (48). To streamline the categories for enhanced comparative analysis of healthcare-seeking behaviors across groups with divergent health statuses, “excellent” and “very good” health conditions were grouped into the first category (good health status), “good” and “fair” health conditions into the second category (medium health status), and “poor” health condition as the third category (poor health status). Columns 1–3 of Table 6 show a considerable positive effect of mobile Internet use on the propensity for self-medication in cases of Health Statuses 1 and 3. However, no notable effects were observed in Health Status 2. Columns 4–6 of Table 6 show a significant influence of mobile Internet use on the preference for PCFs in cases of Health Statuses 1 and 2. Conversely, no significant impact was observed for Health Status 3. Thus, Hypothesis 3 was partially valid.

**Table 6:** Heterogeneous effects.

	Self-medication			PCF choice		
	Health = 1 (1)	Health = 2 (2)	Health = 3 (3)	Health = 1 (4)	Health = 2 (5)	Health = 3 (6)
Mobile Internet use	1.877^***^ (0.251) [0.630]	1.187 (0.111) [0.172]	1.435^***^ (0.136) [0.361]	0.852^**^ (0.077) [−0.160]	0.825^***^ (0.058) [−0.193]	0.895 (0.102) [−0.111]
Constant	0.884 (1.345)	5.171^**^ (0.658)	0.918 (0.890)	2.757^**^ (0.450)	5.419^***^ (0.357)	0.688 (0.639)
Controls	Yes	Yes	Yes	Yes	Yes	Yes
R-squared	0.103	0.059	0.096	0.083	0.090	0.061
Observations (*N*)	634	2,820	1903	6,846	11,365	2,838

Cells represent odds ratios (robust standard errors) [coefficients]. ^*^p < 0.1; ^**^p < 0.05; ^***^p < 0.01. PCF, primary care facility.

## 5 Discussion

In this study, we systematically explored the nexus between mobile Internet use and health-seeking behaviors among individuals, with a special focus on China, a nation undergoing rapid digital transformation. Our analysis transcended the basic examination of the impact of mobile Internet use on health-seeking behaviors as it evaluated the ramifications of the duration of daily mobile Internet use on these behaviors. Subsequently, we conducted an in-depth and rigorous analysis of how mobile Internet use shapes the health-seeking patterns of Chinese citizens using advanced statistical methodologies, including logit, IV, IV-probit, extended regression models, and propensity score matching models. Lastly, the data under consideration were stratified into three distinct categories reflective of health status: good, medium, and poor. This stratification enables a nuanced evaluation of the diverse health effects regarding the influence of mobile Internet use on the health-seeking behaviors of the Chinese population.

Our research findings demonstrated that Chinese mobile Internet users were more predisposed to self-medication when ill. Notably, an extended duration of daily mobile Internet use correlated with an increased propensity for self-medication, suggesting that self-diagnosis and therapy could act as partial substitutes for traditional hospital care. Second, regarding the selection of medical institutions, mobile Internet users exhibited a greater likelihood of opting for high-level hospitals than for community health service institutions. Furthermore, an increase in daily mobile Internet use inversely affected the probability of choosing PCFs. Additionally, our analysis revealed a significant disparity in health outcomes due to the influence of mobile Internet use on health-seeking behaviors. Therefore, discussing these three critical aspects of our study is important.

First, our analysis demonstrated that mobile Internet use significantly increased the tendency for self-medication rather than opting for hospital visits. This observation aligns with the findings of Ma et al. (49), who found that Internet use effectively dismantled barriers to accessing information about diseases, thereby facilitating a discernible shift toward self-care practices as a partial substitute for conventional hospital care. Furthermore, our results align with those of Yang et al. (50), demonstrating that mobile ICTs, particularly healthcare platforms, have emerged as substantial mechanisms to mitigate the escalating demand for hospital services in the Internet plus era. Mobile ICT–based consultations have facilitated the rapid, efficient, and cost-effective provision of medical services (51). Nevertheless, our study extended beyond prior research by delving into the nuanced impact of mobile Internet use on self-medication, moving past the binary approach of “use or no.” We found that frequent mobile Internet use markedly bolstered an individual’s propensity toward self-medication, simultaneously reducing the probability of utilizing formal medical services. This enables residents to utilize medical resources more effectively, alleviating hospital congestion. This is particularly crucial in China, where the healthcare delivery system faces substantial inefficiencies, and it is imperative to reduce healthcare costs (39).

Second, our study revealed that mobile Internet use may diminish the propensity of individuals to seek medical treatment from PCFs. This finding corroborates the observations made by Li et al. (40), which indicated that mobile Internet use could intensify older adults’ perceived risk of diseases, thereby amplifying their inclination to opt for higher-level medical institutions for treatment. Information derived from multiple online sources can result in information overload or distortion, which are factors that are predictive of information-induced anxiety (52, 53), such as the misinterpretation of physical symptoms as indicators of severe medical conditions, coupled with an enduring apprehension of grave illnesses (54, 55). Furthermore, our research revealed that frequent mobile Internet use can significantly diminish an individual’s inclination to seek primary healthcare services. Due to limited professional medical knowledge, individuals are susceptible to being misled by erroneous medical information, thereby facilitating anxiety development. These factors collectively drive residents to seek care at higher-tier hospitals, particularly in China, where such hospitals are perceived as offering superior-quality treatment (56).

Finally, mobile Internet users with good or poor health conditions were more inclined to self-medicate. One possible explanation is that, compared with residents with average health conditions, people with good health conditions might have higher health literacy, making them more competent at navigating online health resources for self-medication. Conversely, those with poor health conditions may have already sought information about their conditions, thus making the mobile Internet a useful tool. However, individuals with average health conditions might not have the same level of engagement or urgency, leading to less reliance on the Internet for self-medication. In contrast, mobile Internet users with good or average health conditions had a reduced tendency to seek treatment at PCFs. One possible explanation is that, compared with residents with poor health conditions, those with good or average health conditions may pay more attention to prevention and early intervention. Our findings align with recent research (57), which suggests that individuals in better health conditions often have a higher level of health literacy and a proactive attitude toward maintaining their well-being. They are more likely to actively use mobile Internet to gather medical information, including hospital ratings and doctors’ specialties, leading to them preferring higher-level hospitals when selecting medical services. Conversely, residents with poor health conditions may already be receiving treatment at higher-level hospitals given their conditions rather than selecting these hospitals based on information obtained from the mobile Internet.

This study has some limitations. First, despite the belief that the effects of mobile Internet use, as estimated from IV regressions, can technically be construed as causal, the inherent cross-sectional nature of this study precludes the identification of the long-term implications of mobile Internet use on health-seeking behaviors. Second, all health metrics used in this study were based on self-reporting—the measurement errors inherent in these indicators are potentially greater than those in objective health measures, such as blood tests. Measurement errors in subjective proxies are generally unavoidable because of the limited time that respondents have to answer survey questions, compounded by the possibility that some participants may not fully disclose their true perceptions or conditions. Third, owing to the lack of requisite data, we could not explicitly investigate the mechanisms underlying the influence of mobile Internet use on health-seeking behaviors. Future research would benefit substantially from incorporating large-scale longitudinal data encompassing detailed and comprehensive health measures. Additionally, exploring the mediating role of various channel variables could substantially enhance our understanding of the intricate relationship between mobile Internet utilization and individuals’ health-seeking behaviors.

The results of this study have significant implications for policymaking. As emphasized by Chen et al. (58), the swift progression of contemporary media technologies, particularly the widespread adoption of smartphones and mobile Internet, can significantly alter individuals’ medical decision-making processes, which are influenced by the availability of additional online health information. The advent of mobile Internet technology has significantly reduced barriers to acquiring knowledge about diseases, diminished disparities in accessing health information, and induced a notable substitution effect. This effect is characterized by a trend in which self-medication increasingly supplements the need for traditional hospital care. However, the credibility, accuracy, and reliability of health information obtained from the Internet are of great concern (59). Health information on the Internet may lack the accuracy and rigor presented in textbooks or scholarly journals (60), consequently introducing ambiguities into the decision-making process and obscuring individuals’ understanding of medical conditions. For instance, a study by Farnood et al. (61) has revealed that the increased use of online health forums and mobile health apps has led to a higher incidence of self-diagnosis and self-treatment among patients, which can both positively and negatively impact healthcare outcomes. Similarly, Saba et al. (62) have demonstrated that while mobile health technologies can improve health literacy and patient engagement, they also pose risks related to misinformation and self-medication without professional guidance. Consequently, individuals with poor health conditions who ideally seek professional medical care in hospitals may resort to self-medication, whereas those with good health conditions who might be adequately served by PCFs may prefer seeking services at larger hospitals. Governmental authorities must enact policies that ensure the involvement of public health practitioners and healthcare professionals in creating, distributing, and appraising Web-based health and medical information, thereby steering patients toward making informed, individualized healthcare decisions. This approach is essential for realizing structured diagnosis and treatment, economizing on expenses, and significantly enhancing the efficiency and quality of services.

## Data availability statement

Publicly available datasets were analyzed in this study. This data can be found here: http://www.isss.pku.edu.cn/cfps/index.htm.

## Ethics statement

The studies involving humans were approved by Biomedical Ethics Committee, Peking University IRB00001052-14010. The studies were conducted in accordance with the local legislation and institutional requirements. Written informed consent for participation in this study was provided by the participants’ legal guardians/next of kin.

## Author contributions

CL: Conceptualization, Data curation, Formal analysis, Funding acquisition, Investigation, Methodology, Project administration, Resources, Software, Supervision, Validation, Writing – original draft, Writing – review & editing. HL: Conceptualization, Data curation, Investigation, Software, Validation, Visualization, Writing – review & editing.
